# A fixation-weighted null model for the proportion of sex chromosome-autosome fusions

**DOI:** 10.17912/micropub.biology.002109

**Published:** 2026-08-31

**Authors:** Sarah Schmalz, Heath Blackmon

**Affiliations:** 1 Department of Biology, Texas A&M University

## Abstract

Recent null models for sex-autosome fusions have often relied on occurrence probability alone. However, evolutionary expectations must account for fixation probability. Under neutrality, fixation probability is inversely proportional to chromosomal population copy numbers, which vary across autosomes, X chromosomes, and Y chromosomes. We extend the original fusion model by weighting each fusion class by its relative neutral fixation probability. This updated model predicts substantially higher proportions of fixed SA-fusions, establishing a more biologically realistic null hypothesis. We provide an updated R function in the R package evobiR and demonstrate its impact by re-evaluating empirical fusion patterns in *Drosophila* and *Habronattus* .

**Figure 1. Expected conditional probability of fixed sex-autosome (SA) fusions as a function of diploid autosome count:** Plots show four sex chromosome systems (XO, XY, XXY, XYY). The dashed lines represent the occurrence probability null model (Anderson et al., 2020), where all non-homologous chromosomes are equally likely to fuse. The occurrence baseline is insufficient for evaluating real-world phylogenetic data, as comparisons rely on variants that successfully survive genetic drift rather than mutation occurrence alone. The solid lines represent the fixation-weighted probability model. The horizontal dotted line marks the P = 0.25 threshold, highlighting the divergence across autosome counts between the two models as detailed in the text. All calculations assume equal mutation rates in males and females ( *μ _d_* =0.5).

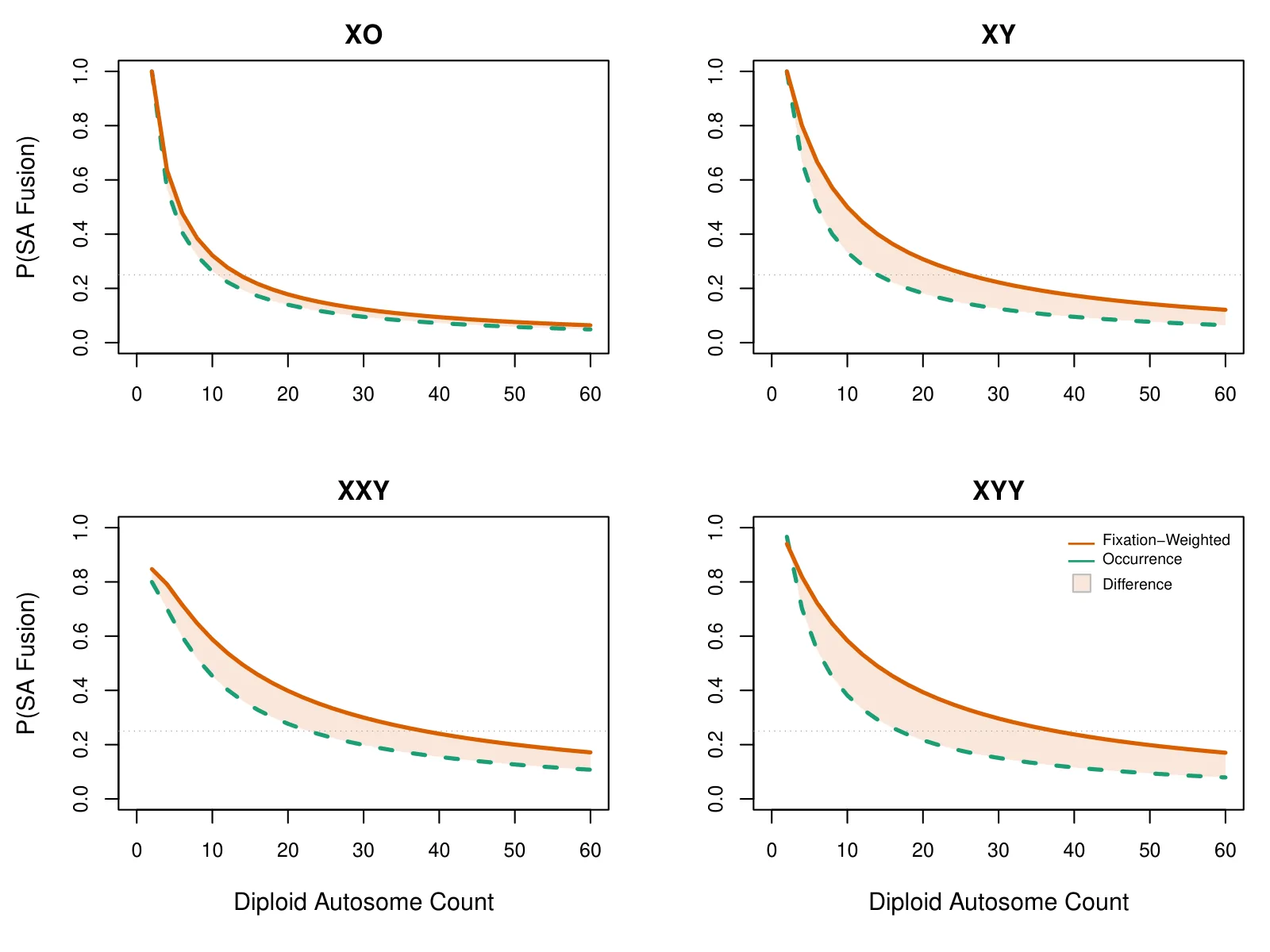



## Description

Chromosomal fusions are a primary mechanism of karyotype evolution and play a central role in the formation of neo-sex chromosomes (White, 1977; Blackmon et al., 2019). Anderson et al. (2020) derived closed-form expressions for the probability that a random chromosomal fusion joins a sex chromosome and an autosome (SA-fusion), two autosomes (AA-fusion), or two sex chromosomes (SS-fusion), under a null model where all non-homologous chromosomes are equally likely to participate in a fusion. These equations account for chromosome number, sex chromosome system, and unequal contributions of the two sexes. Using this occurrence-based null model, Anderson et al. (2020) found that *Drosophila* exhibited significantly fewer SA-fusions than expected (observed proportion 0.155, expected 0.43), suggesting that SA-fusions may be selected against in this clade. Conversely, they observed a significant excess of SA-fusions in the jumping spider genus *Habronattus* (observed proportion 0.8, expected 0.194), hypothesizing that positive selection drives their accumulation to resolve sexual antagonism.

However, the occurrence of a fusion as a new mutation and its fixation in a population are governed by different processes. Under neutrality, the probability that a single new mutation reaches fixation is the reciprocal of the number of copies of that chromosome in the population (Ewens, 2004). With N diploid individuals and an equal sex ratio, autosomes are present in 2N copies, X chromosomes in 3N/2 copies (two per female, one per male), and Y chromosomes in N/2 copies (one per male). Consequently, a fusion arising on the Y chromosome is approximately 4 times more likely to fix than an equivalent autosomal fusion, and an X-linked fusion is approximately 4/3 times more likely (Charlesworth et al., 1987). To incorporate these differences, we extend the Anderson et al. (2020) model by assigning relative fixation weights (w) to each fusion class:

$$w_{A}=1,\;w_{X}=\frac{4}{3},\;w_{Y}=4$$

We first compose the formulae for the occurrence proportion of fusions that are expected to join an autosome and a sex chromosome in the homogametic and heterogametic sex (i.e., P(SA|hom) and P(SA|het)). These sex-specific occurrence proportions are derived from the component terms of Anderson et al. (2020) Equation 2.2 for P(SS) and Equation 2.3 for P(AA) via the complement rule:

$$P\left(SA|hom\right)=1-\frac{D_{a}\left(D_{a}-2\right)}{D_{d}\left(D_{d}-2\right)}-\frac{4X_{s}\left(X_{s}-1\right)}{D_{d}\left(D_{d}-2\right)}$$

$$P\left(SA|het\right)=1-\frac{D_{a}\left(D_{a}-2\right)}{D_{s}\left(D_{s}-2\right)}-\left[\frac{X_{s}\left(X_{s}-1\right)}{D_{s}\left(D_{s}+X_{s}-1\right)}+\frac{Y\left(Y-1\right)}{D_{s}\left(D_{s}+Y-1\right)}\right]$$

In the homogametic sex, all sex chromosomes are X, so all SA-fusions are XA-fusions. In the heterogametic sex, SA-fusions are partitioned between XA and YA in proportion to the number of X and Y chromosome arms available:

$$P\left(\text{XA}\right)=\mu_{d}\cdot P\left(\text{SA}|\text{hom}\right)+\left(1-\mu_{d}\right)\cdot P\left(\text{SA}|\text{het}\right)\cdot \frac{X_{s}}{X_{s}+Y}$$

$$P\left(\text{YA}\right)=\left(1-\mu_{d}\right)\cdot P\left(\text{SA}|\text{het}\right)\cdot \frac{Y}{X_{s}+Y}$$

where 𝜇 _d_ is the proportion of fusions originating in the homogametic sex, X _s_ and Y are the counts of X and Y chromosomes in the heterogametic sex:

$$P\left(\text{XX}\right)=\mu_{d}\cdot P\left(\text{SS}|\text{hom}\right)+\left(1-\mu_{d}\right)\cdot \frac{X_{s}\left(X_{s}-1\right)}{D_{s}\left(D_{s}+X_{s}-1\right)}$$

$$P\left(\text{YY}\right)=\left(1-\mu_{d}\right)\cdot \frac{Y\left(Y-1\right)}{D_{s}\left(D_{s}+Y-1\right)}$$

where D _s_ is the diploid chromosome number in the heterogametic sex, and P(SS) is the SS-fusion occurrence probability from Anderson et al. (2020, Equation 2.2).

The fixation-weighted proportion of SA-fusions among all fusions is then:

$$P_{\text{fix}}\left(\text{SA}\right)=\frac{w_{X}\cdot P\left(\text{XA}\right)+w_{Y}\cdot P\left(\text{YA}\right)}{w_{A}\cdot P\left(\text{AA}\right)+w_{X}\left[P\left(\text{XA}\right)+P\left(\text{XX}\right)\right]+w_{Y}\left[P\left(\text{YA}\right)+P\left(\text{YY}\right)\right]}$$

The fixation-weighted model predicts markedly higher proportions of SA-fusions than the occurrence model across all sex chromosome systems and autosome counts (Figure 1). For a standard XY system, the occurrence-based P(SA) does not drop below 0.25 until the diploid autosome count reaches 16 (Anderson et al., 2020). Under the fixation-weighted model, this threshold is not reached until the diploid autosome count reaches 28. The effect is driven primarily by Y-autosome fusions: because the Y is present in so few copies, YA-fusions that do occur have a disproportionately high probability of reaching fixation.

This has direct implications for interpreting the *Drosophila* results of Anderson et al. (2020). *Drosophila* species in their dataset had diploid numbers ranging from 6 to 12, corresponding to diploid autosome counts of 4 to 10 for an XY system. Across this range, the fixation-weighted expected proportion of SA-fusions is 0.50–0.80, compared to 0.33–0.67 under the occurrence model. The observed proportion of 0.155 (credible interval 0.12–0.22) therefore falls even further below the neutral expectation under the fixation-weighted model than under the occurrence model alone. This strengthens the conclusion that SA-fusions are disfavored in *Drosophila* , consistent with the hypothesis that achiasmatic meiosis in males amplifies the deleterious consequences of SA-fusions by exposing the entire neo-Y to degenerative forces (Anderson et al., 2020).

Conversely, applying the fixation-weighted model also recalibrates our understanding of clades with an apparent excess of SA-fusions. Anderson et al. (2020) highlighted empirical data from the jumping spider genus *Habronattus* (XXO sex chromosome system, typical diploid autosome count of 26), which exhibits a high frequency of SA-fusions (observed proportion 0.8) hypothesized to resolve sexual antagonism (Maddison and Leduc-Robert, 2013). Under the occurrence model, the expected probability of an SA-fusion is 0.194. However, under the fixation-weighted model, the baseline neutral expectation rises by 25% to 0.243. Following the methodology of Anderson et al. (2020), we calculated the exact empirical p-value for the observed 8 out of 10 fusions using a multinomial distribution. The resulting statistical “excess” (0.8 observed vs. 0.243 expected) is less extreme than previously estimated (p = 3.33 ⋅ 10 ^-4^ vs. p = 6.19 ⋅ 10 ^-5^ ). Although our refined framework recalibrates the specific null probabilities, the qualitative conclusion of a significant SA-fusion excess in *Habronattus* remains robust, mirroring the *Drosophila* analysis.

More broadly, the fixation-weighted model provides a more biologically realistic null expectation for comparative studies of karyotype evolution. Researchers comparing observed fusion patterns to null expectations should consider whether fixation probability, rather than occurrence alone, is the appropriate baseline. This is especially relevant in clades with low chromosome numbers, where the discrepancy between the two models is greatest. We have implemented the fixation-weighted model as the function pSAF in the R package evobiR (Jonika et al., 2023). Though our method is described in terms of male heterogametic systems, the function also accommodates XX/XY and ZZ/ZW-type systems, and allows users to toggle between occurrence and fixation-weighted calculations. 

## Methods

All equations were implemented in R (version 4.4.1) (R Core Team, 2024). The function pSAF is available in the evobiR package ( https://github.com/coleoguy/evobir ) (Jonika et al., 2023). Fixation weights were derived as the inverse of the effective number of chromosomal copies per generation (2N for autosomes, 3N/2 for X chromosomes, N/2 for Y chromosomes), assuming an equal sex ratio and neutrality. The population size parameter N cancels when computing proportions.

The authors utilized the LLM Google Gemini as a collaborative writing and editing tool. The AI was used to assist in the structuring of the manuscript, refining clarity of the text, and ensuring compliance with journal formatting guidelines. All mathematical derivations, scientific conclusions, and data analyses were performed solely by the authors.
